# Successful percutaneous coronary intervention for an in-stent chronic total occlusion in a patient with dextrocardia: a case report

**DOI:** 10.1186/s12872-017-0712-1

**Published:** 2017-11-16

**Authors:** Johannes Wild, Tommaso Gori, Thomas Münzel, Philip Wenzel

**Affiliations:** 1 Center for Cardiology – Cardiology I, University Medical Center Mainz, Langenbeckstrasse 1, 55131 Mainz, Germany; 2 German Center for Cardiovascular Research (DZHK) – Partner site Rhine-Main, University Medical Center Mainz, Langenbeckstrasse 1, 55131 Mainz, Germany; 3 Center for Thrombosis and Hemostasis, University Medical Center Mainz, Langenbeckstrasse 1, 55131 Mainz, Germany

**Keywords:** Dextrocardia, Coronary artery disease, Chronic total occlusion

## Abstract

**Background:**

Percutaneous coronary interventions of chronic total occlusion represent one of the most challenging issues in interventional cardiology. A Caucasian patient with dextrocardia presented with an in-stent chronic total occluded right coronary artery, a constellation which has not been described previously in the literature.

**Case presentation:**

A 69-year-old man with pre-known situs inversus totalis and a long history of coronary artery disease with multiple interventions and stent-implantations presented to our department suffering from episodes of chest pain under exercise. A coronary angiogram showed a completely occluded right coronary artery in the area of a drug-eluting stent which had been implanted eight years before. We found collaterals from the left coronary artery system and signs of calcification, so the lesion was classified as chronic total occlusion and the recanalization using an antegrade wire escalation technique successfully performed.

**Conclusion:**

This is the first reported case of an in-stent chronic total occlusion in situs inversus with dextrocardia. Our experience in this case demonstrates the feasibility of recanalization of in-stent chronic total occlusions even in the rare setting of this congenital cardiac malposition combined with severe coronary artery disease.

## Background

Dextrocardia is a congenital malposition which occurs in only 1 of 10,000 humans [1]. Atherosclerosis in general occurs as often in patients with dextrocardia as in the general population, so, given the rarity of the malposition, chronic total coronary occlusions (CTO) seen in this special population are extremely rare. We now report the case of a patient with dextrocardia and coronary three-vessel disease with the successful ad-hoc recanalization of an in-stent chronic total occluded right coronary artery.

## Case presentation

A 69-year-old man with known situs inversus totalis and dextrocardia presented to our department due to frequent episodes of chest pain under minimal exercise (Canadian Cardiovascular Society grading of angina pectoris Class III). The patient presented with arterial hypertension, hypolipoproteinemia and nicotine abuse (45 pack years) as cardiovascular risk factors and a known history of a complex coronary three-vessel disease. About eight years ago, the patient was admitted to a hospital abroad with an acute coronary syndrome and the right coronary artery (RCA) was treated with a drug-eluting stent. Four years ago the patient had reported typical chest pain and the left main was treated with an everolimus-eluting stent as well as the proximal left anterior descending artery (LAD) and left circumflex artery (RCX) dilated in kissing balloon technique. At this previous coronary angiography, the RCA had not shown any de-novo stenosis or in-stent restenosis.

**Fig. 1:**
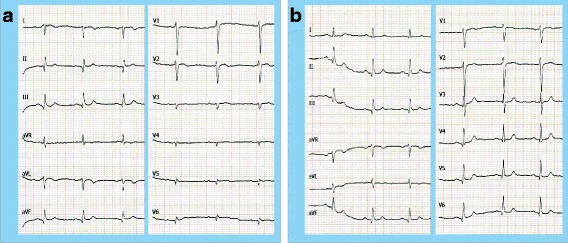
Electrocardiogram in dextrocardia (25 mm/s, 10 mm/mV). **a** Conventional placement of the ECG leads with the typical findings of dextrocardia: right axis deviation, positive QRS complexes (with upright P and T waves) in aVR, ‘global negativity’ (inverted P wave, negative QRS, inverted T wave) in I and absent R-wave progression in the chest wall leads. **b** Mirror inverted placement of the ECG leads on the right side of the chest and reversing the left and right arm leads

Now, the patient presented with typical chest pain and dyspnea under minimal exercise which had increased during the previous months. The patient denies palpitations or other symptoms. In the physical examination, he did not show any pathologies. The heart sounds could be auscultated on the right side of his chest without any murmurs, no peripheral edema could be seen. An ECG with the usual placement of the electrodes showed typical signs of dextrocardia: right axis deviation, positive QRS complexes (with upright P and T waves) in aVR, ‘global negativity’ (inverted P wave, negative QRS, inverted T wave) in I and absent R-wave progression in the chest leads (Fig. 1a). As recommended in the literature, an additional electrocardiogram was recorded after placing the precordial leads in a mirror-image position on the right side of the chest and reversing the left and right arm leads. As sign of the known former myocardial infarction Q-waves in II/III/aVF were present (Fig. 1b).

The laboratory findings did not show any pathologic results, especially the cardiac markers troponin and creatine kinase were normal. A transthoracic echocardiography at rest revealed a regular left ventricular function with apical hypokinesia which had already been described in the previous echocardiography. No abnormalities of the valves were seen. A stress echocardiogram by treadmill could not be performed due to severe arthralgia and significant dyspnea. Because of the typical symptoms and the complicated coronary three vessel-disease with a history of percutaneous coronary interventions and stent-implantations, the indication for a coronary angiography was given.

**Fig. 2:**
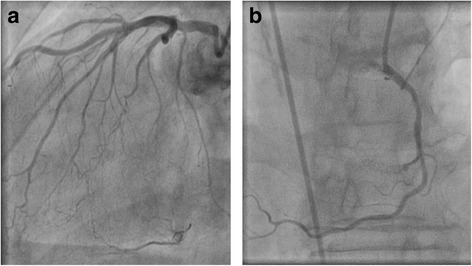
Coronary angiogram of RCA-in-stent CTO in dextrocardia. **a** In-stent RCA-CTO (angulation RAO 2.1°, CRAN 28.1°). **b** Left coronary artery with collaterals to the right coronary artery (angulation LAO 30°, CRAN 0°)

The vascular access was established by the right femoral artery and a 6French Terumo® sheath inserted. Due to known dextrocardia, we exceptionally preferred the femoral access over the radial access route in this specific case. The coronary angiography showed a good result after the previous left main stenting and RCX/LAD percutaneous coronary intervention (Fig. 2a). In contrast, the RCA was completely occluded in segment two right in the area of the stent that was implanted ten years before (Fig. 2b). We found collaterals from the left coronary artery system and signs of calcification (Fig. 2a), so the diagnostically criteria of a CTO were fulfilled. Collaterals were grade I – II according to Rentrop classification. The Japanese-CTO-score (J-CTO-score) [2] which describes the complexity of the lesion was 3 (due to the occlusion length of more than 20 mm, bending and the present calcification), indicating a very difficult lesion. Ventriculography showed a regular-sized left ventricle with good systolic function. Because of the present symptoms of the patient and evidence for vital myocardium by echocardiography, an ad-hoc revascularization of the CTO was attempted.

**Fig. 3:**
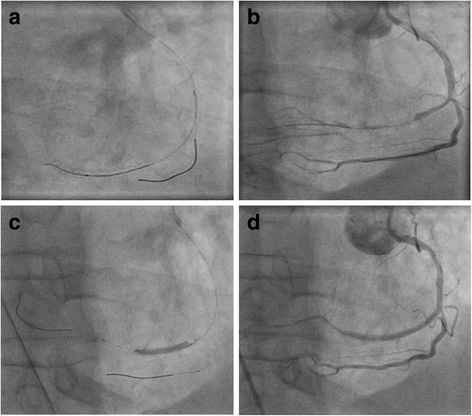
Coronary angiogram of in-stent CTO-PCI in dextrocardia. **a** Placement of a buddy wire (Abbott® BMW-CW) in a right-ventricular branch and an Abbott Hi-Torque Progress wire for recanalization (angulation RAO 29.30° CAUD 0.2°). **b** Successful recanalization of the RCA-CTO. **c** PTCA with an Abbott NC Trek® 2.5/20 mm balloon. **d** Final result with complete recanalization and TIMI III flow after implantation of two everolimus eluting stents (Abbott Xience Pro® 2.5/23 mm and Xience Pro 2.75/23 mm)

A Cordis 6F SRC® no-torque guidance catheter was used and the standard antegrade wire escalation technique attempted. A buddy-wire (Abbott® BMW-CW) was inserted in a right-ventricular branch of the RCA. A Terumo Finecross® microcatheter was inserted with the help of a BMW-wire which was exchanged to an Abbott Hi-Torque Progress 200 T® which allowed the successful recanalization (Fig. 3a/b). Due to rapid guidewire success and clear demarcation of the CTO segment, we refrained form using a second arterial access to visualize collaterals. Balloon angioplasty was performed with Terumo Tazuna® 1.25/10 mm, Boston Scientific Maverick® 1.5/20 mm and Abbott NC Trek® 2.5/20 mm (eight insufflations with 16 bar maximum, Fig. 3c). Two everolimus eluting stents (Abbott Xience Pro® 2.5/23 mm with 18.0 bar and Xience Pro 2.75/23 mm with 14 bar) were successfully implanted with very good angiographic result (Fig. 3d).

The patient was loaded with clopidogrel for dual platelet aggregation inhibition in addition to his premedication with aspirin. Overall, 254 ml of contrast agent were used during this procedure. Even though the J-CTO-score (indicating the complexity of the lesion) was 3 and thus higher than the average at our department (which is 2,46), the lesion could be treated faster (116 min vs 126 min) and with lower fluoroscopy time (23 min vs 28,3 min) than the average at our department.

After the procedure, there was no clinical sign for pericardial effusion and no significant elevation of cardiac markers. The patient reported no symptoms and was discharged the next day after the procedure.

## Discussion and conclusions

According to the current guidelines of the European Society of Cardiology, a CTO lesion is defined as a coronary TIMI flow of zero for at least three months [3]. Since the time of occlusion is – as in the presented case - not always known, the EuroCTO club published different levels of certainty if an occlusion can be classified as CTO. Due to this classification [3], the presented case would be judged with the level of certainty as possible or undetermined. The occlusion showed a TIMI 0 flow and the angiographic anatomy was suggestive of long-standing occlusion with collateral development and no contrast staining. The Japanese CTO register [2] suggests for patients who did not undergo previous catheterization the definition as a CTO based on the patient’s clinical history and the angiogram, especially the presence of severe calcification as diagnosed in this patient is seen as a diagnostic criteria for CTO.

Due to growing experience with the percutaneous interventional approach for this kind of lesions and new technical features, the procedural success rates for CTO-revascularizations have significantly improved within the last years and is now considered within the 90% range [4]. In experienced centers, it can nowadays routinely be performed by radial approach [5]. Despite these improvements, the lesion subset of in-stent CTOs has always been associated with lower procedural success rates (63% to 71%) [6] and still remains challenging for interventional cardiologists. In general, coronary chronic total occlusions due to in-stent restenosis are quite common, representing 5% to 25% of all CTO percutaneous coronary interventions. Regarding the presented case, given the rarity of dextrocardia, this is only the third report of a successful percutaneous CTO treatment in a patient with dextrocardia [7, 8] and the first report of the diagnosis and successful treatment of an in-stent CTO in this rare congenital condition.

The risk for structural heart disease is elevated in patients with situs inversus and dextrocardia, anomalies which are diagnosed more frequently in these patients are ventricular septal defect, transposition of the great arteries, double outlet right ventricle and atrial appendage juxtaposition [9]. On the contrary, the risk for atherosclerosis is as high in patients with dextrocardia as in the general population [10].

Our experience in this case demonstrates the feasibility of recanalization of an in-stent CTO even in the rare setting of this congenital cardiac malposition.

## Abbreviations

CTO: Chronic total occlusion
LAD: Left anterior descending artery
RCA: Right coronary artery
RCX: Left circumflex artery
